# A case of Kernohan-Woltman notch phenomenon caused by an epidural hematoma: the diagnostic and prognostic value of PET/CT imaging

**DOI:** 10.1186/s12883-022-02965-y

**Published:** 2022-11-10

**Authors:** Yuliang Lin, Alan Chen-Lung Chou, Xiangming Lin, Zhende Wu, Qichao Ju, Yuexuan Li, Zulong Ye, Bo Zhang

**Affiliations:** 1 Department of Neurosurgery, Xiamen Chang Gung Hospital, No. 123, Xiafei Road, Haicang District, Xiamen, China; 2 Department of Rehabilitation Medicine, Xiamen Chang Gung Hospital, No. 123, Xiafei Road, Haicang District, Xiamen, China

**Keywords:** Kernohan-Woltman notch phenomenon, Traumatic brain injury, Cerebral peduncle, Ipsilateral hemiparesis, Functional recovery, Positron emission tomography

## Abstract

**Background:**

Kernohan-Woltman notch phenomenon (KWNP) classically occurs when a lesion causes compression of the contralateral cerebral peduncle against the tentorium, resulting in ipsilateral hemiparesis. It has been studied clinically, radiologically and electrophysiologically which all confirmed to cause false localizing motor signs. Here, we demonstrate the potential use of fluorine-18 fluorodeoxyglucose (18 F-FDG) positron emission tomography/computed tomography (PET/CT) to identify KWNP caused by an epidural hematoma.

**Case presentation:**

A 29-year-old male patient post right-sided traumatic brain injury presenting with persistent ipsilateral hemiparesis. Patient underwent decompressive craniotomy and intracranial hematoma evacuation. Brain magnetic resonance imaging in the postoperative period showed a subtle lesion in the left cerebral peduncle. PET/CT was performed to exclude early brain tumor and explain his ipsilateral hemiparesis. PET/CT imaging demonstrated a focal region of intense 18 F-FDG uptake in the left cerebral peduncle. Throughout the treatment in outpatient neurorehabilitation unit, the patient exhibited a gradual recovery of his right hemiparesis.

**Conclusion:**

In our case report, for the first time, PET/CT offered microstructural and functional confirmation of KWNP. Moreover, our case suggests that 18 F-FDG PET/CT may serve as an important reference for the probability of functional recovery.

## Background

Kernohan-Woltman notch phenomenon (KWNP) is a false localizing sign due to the compression of the contralateral cerebral peduncle, which may still cause diagnostic and clinical confusion [1]. It was first described in a patient with ipsilateral hemiparesis to a brain tumor in 1929 [2]. In 1990 magnetic resonance imaging (MRI) was performed by Cohen et al. [3] which offered radiographic verification of KWNP. Here, we present the first case of KWNP confirmed by fluorine-18 fluorodeoxyglucose (18 F-FDG) positron emission tomography/computed tomography (PET/CT).

## Case presentation

**Fig. 1:**
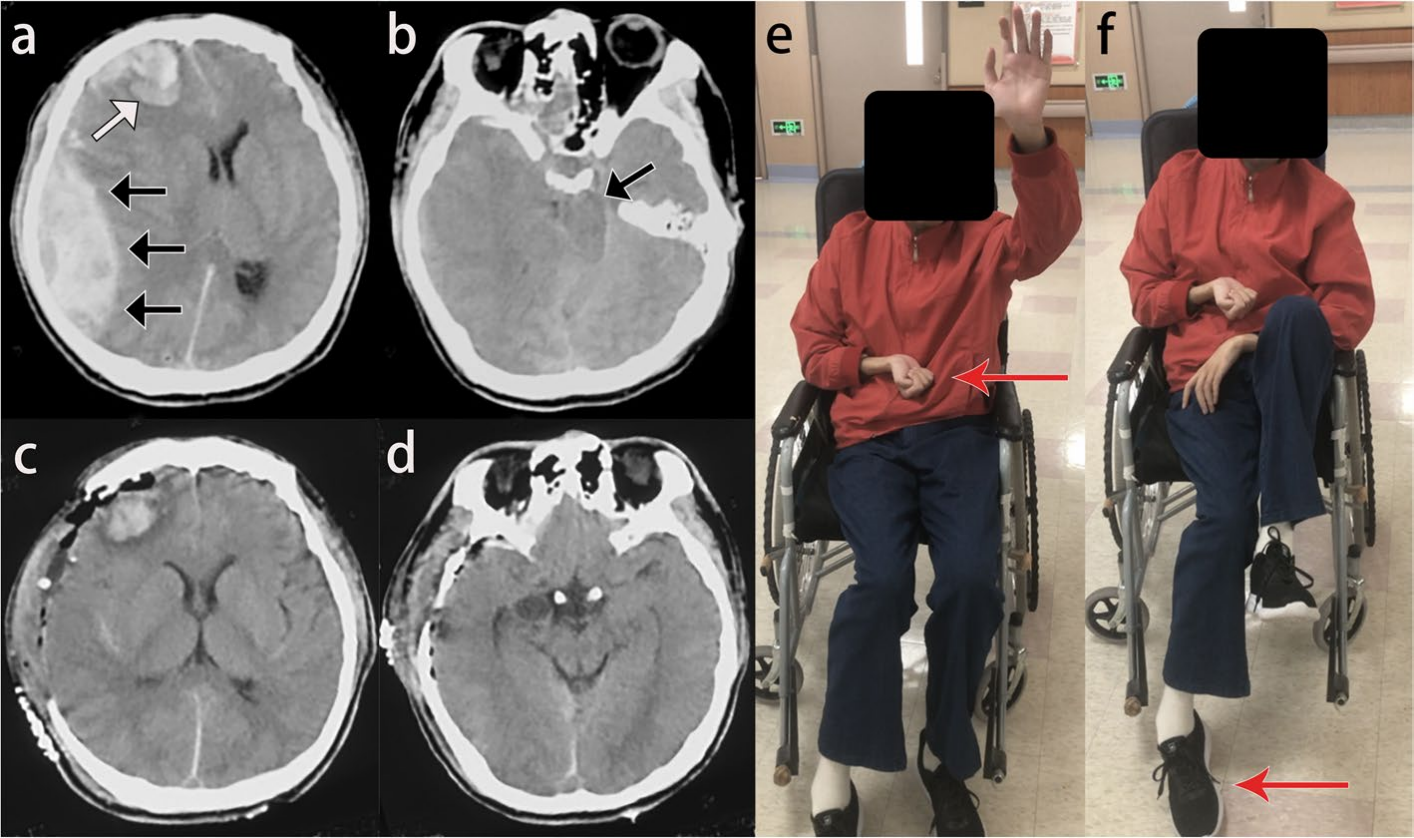
**a** Preoperative computed tomography (CT) scan revealed a right epidural hematoma (black arrows) and frontal lobe contusion (white arrow) with significant right-to-left midline shift. **b** A descending transtentorial herniation and total obliteration of all basal cerebrospinal fluid spaces by the displacement of brain can be seen. Note the left-mesencephalon notching against the tentorial edge (black arrow). **c, d** Postoperative CT scans. **e, f** The patient’s right hemibody strength was 2/5 (red arrows), indicating a right-sided hemiparesis

A 29-year-old male arrived at the outpatient department with complaint of persistent right-sided hemiparesis. 2 years ago, he sustained a traumatic brain injury (TBI) and was admitted in the local emergency department, head CT revealed a right epidural hematoma and frontal lobe contusion (Fig. 1a, b). Patient underwent decompressive craniotomy and epidural hematoma evacuation (Fig. 1c, d). In the postoperative period he was noted to have a persistent right-sided hemiparesis which prompted him transfer to our institution for further management (Fig. 1e, f).

**Fig. 2:**
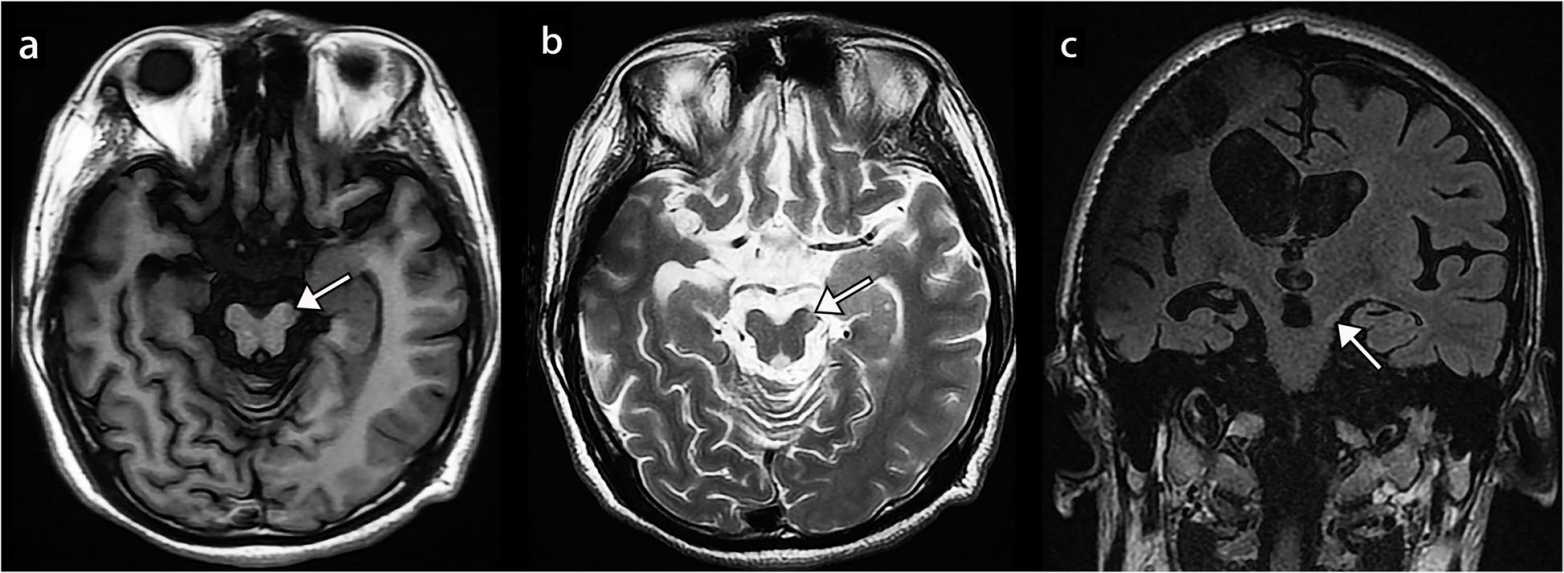
Magnetic resonance imaging displayed a subtle lesion (white arrow) in the left cerebral peduncle probably associated with the Kernohan-Woltman notch phenomenon. **a** Axial T1-weighted sequence. **b** Axial T2-weighted sequence. **c** Coronal FLAIR sequence also showed a right frontal lobe encephalomalacia and a moderate communicating hydrocephalus

**Fig. 3:**
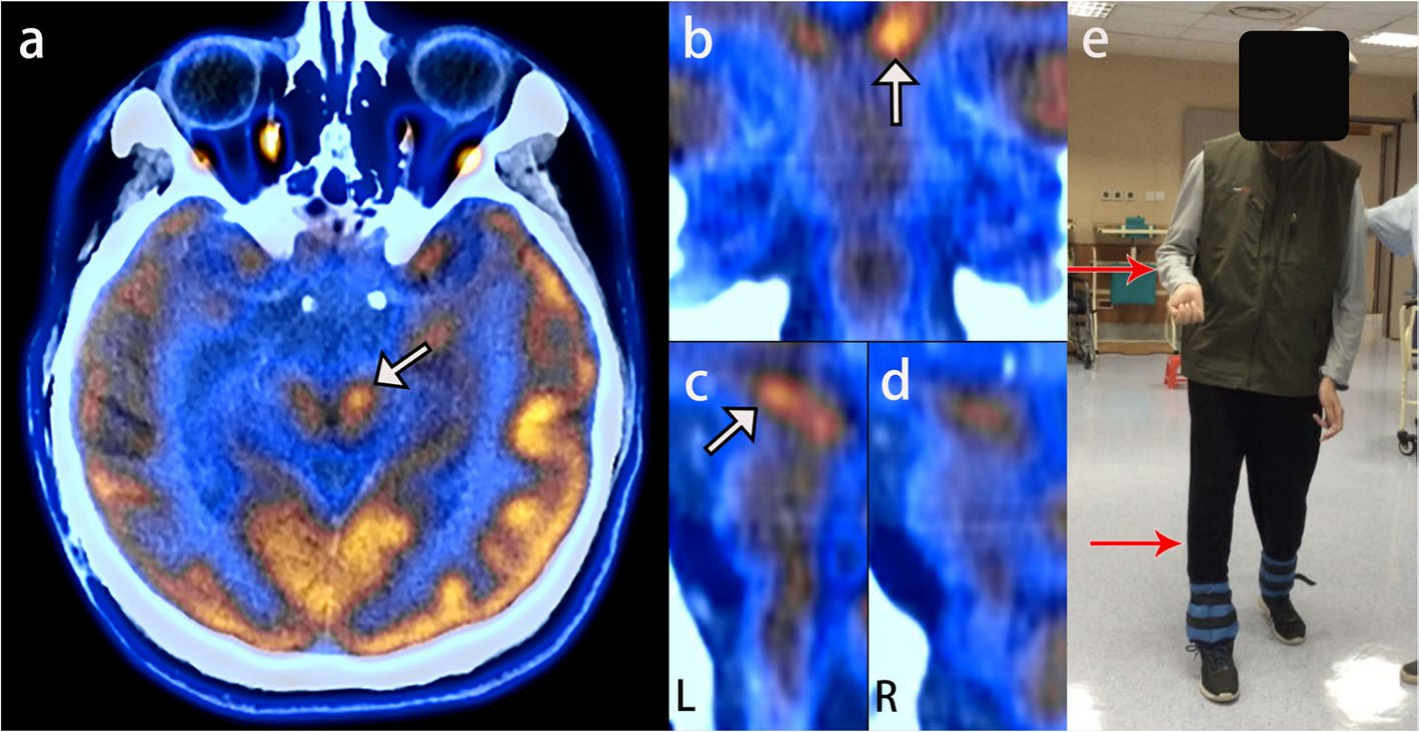
Patient was food-deprived 6 h before positron emission tomography/computed tomography (PET/CT) scanning. Emission data were acquired for 50 min after an intravenous bolus injection of 292 MBq fluorine-18 fluorodeoxyglucose (18 F-FDG). The mean standardized uptake value of each cerebral peduncle was measured on fused PET and CT image. PET/CT imaging demonstrated a focal region of intense 18 F-FDG uptake in the left cerebral peduncle (white arrow). **a** Axial view. **b** Coronal view. **c** Left parasagittal view. **d** In the right parasagittal view of PET/CT imaging at the level of the brainstem, no intense 18 F-FDG uptake can be seen above the pyramidal decussation. **e** The patient had right-sided muscular rigidity (red arrows) and was able to walk unassisted after rehabilitation treatment

On admission, the patient maintained normal consciousness and brisk reaction of both pupils. Neurological examination revealed deep tendon reflexes asymmetry ( + + on the left, +++~++++ on the right) and a positive right-sided Babinski sign. His right-sided strength was 2/5. Paradoxically, he had no neurologic symptoms and signs related to the right hemisphere. Brain MRI demonstrated a subtle T1 hypointense and T2 hyperintense lesion in the left cerebral peduncle indicating KWNP (Fig. 2). The MRI also showed a right frontal lobe encephalomalacia and a moderate communicating hydrocephalus. 18 F-FDG PET/CT was performed to exclude early brain tumor and demonstrated neuronal function and activity of the injured brain. The standardized uptake value were summarized from 3 random fields of each cerebral peduncle. There was a higher 18 F-FDG uptake in the left cerebral peduncle (Fig. 3a-c) compared to the contralateral side (Fig. 3d) (7.74 ± 0.68 vs. 5.87 ± 0.7, *p* < 0.05).

The patient underwent a V-P shunt and was transferred to outpatient neurorehabilitation unit. He exhibited a gradual recovery of his right hemiparesis. At the last follow-up post 50 months injury, he still had hypermyotonia and tendon hyperreflexia in the right extremities. However, his right-sided strength had improved to 4/5. Patient was able to walk unassisted and was discharged from our institution (Fig. 3e).

## Discussion and conclusions

In the pre-MRI era, KWNP was one of the most widely accepted theories for explaining ipsilateral hemiparesis [4]. It has been well studied clinically, radiologically and electrophysiologically in recent years [5]. KWNP classically occurs when a lesion causes compression of the contralateral cerebral peduncle against the tentorium, resulting in disruption of corticospinal tract fibers [1]. The combination of mechanical and ischemic impairment ultimately results in dysfunction of the contralateral descending motor system above the medullary decussation, manifesting as a hemiparesis ipsilateral to the primary lesion [6]. MRI features of KWNP typically include T2 and FLAIR hyperintensity and T1 hypointensity within the contralateral cerebral peduncle [3, 4]. However, several KWNP patients experience persistent neurological dysfunction with no signal change on conventional MRI [7]. It is urgent to develop advanced imaging modalities to detect and better understand KWNP.

PET/CT is a radiographic technique primarily used for diagnosis and staging of glioma [8, 9]. In our case no brain tumor was found. On the other hand, PET/CT has also been used to investigate the microstructural and functional changes of the injured brain [10]. PET/CT signal changes were seen in many brain regions, including the midbrain [11, 12]. The tracer 18 F-FDG is a glucose analogue used to investigate glucose metabolism in vivo, and glycolytic pathways are upregulated and result in a large increase in 18 F-FDG uptake during brain injury [13]. In our case, increased 18 F-FDG uptake was detected in the left cerebral peduncle displaying midbrain function alteration which offered microstructural and functional confirmation of KWNP. Therefore, PET/CT can be used as a more accurate auxiliary diagnostic technique for the diagnosis of KWNP than conventional MRI.

There has been little description of long-term outcome for patients who develop KNWP. Zhang et at. [1] reviewed 22 TBI KWNP patients, 77% showed improvement in motor function, with 27% having resolved neurology. The long-term persistence of midbrain MRI alterations is incompletely understood. Signal changes were seen even when MRI was performed 9 months after injury [14]. Oster et al. [15] reported that the T2 hyperintense lesion on a KWNP patient can resolve with rehabilitation. Moon et al. [16] described that a lack of MRI signal change may correlate with a better functional prognosis. In our case, a subtle MRI change within the left cerebral peduncle can still be detected 35 months post injury. It may imply that patients with long-term cerebral peduncle lesion take longer to rehabilitate.

PET/CT facilitates the recognition of the pathophysiological mechanisms underlying KWNP which may provide prognostic information regarding potential for functional recovery. In traumatic brain, cerebral glucose metabolism significantly associates with neuronal function and activity of cells including neurons and glial cells [12, 17]. Von Leden et al. [18] demonstrated that the increased 18 F-FDG uptake is caused by an elevated glial cell response. Degenerated axons can be replaced by reactive gliosis, which is primarily the proliferation of astrocytes to form a scar in the later stage of brain injury [19]. The presence of radiological lesions, which correspond to the area of demyelination and reactive gliosis within the corticospinal tract, may predict a low probability of functional recovery [20]. It is possible that the increased 18 F-FDG accumulation in our patient was induced by neuroinflammation following the compression of the contralateral cerebral peduncle. Despite the 50-month-long hemibody weakness and tendon hyperreflexia, our patient was able to walk unassisted after rehabilitation treatment, seemingly implying that the long-term motor deficit of KWNP patient is still reversible.

Although PET/CT imaging provides exquisite sensitivity when compared with CT or MRI, it is not always practical to use, particularly in the emergency setting of severe TBI. The application of PET/CT may be hindered by its high cost and time-consuming. Due to the lack of PET/CT re-examination after rehabilitation treatment, whether there are significant lesion size changes in the cerebral peduncle or not remains unknown. In the future, a statistical parametric mapping analysis is required to further corroborate the brain metabolic alterations and the pathophysiological mechanisms of KWNP.

In conclusion, PET/CT can be helpful in differentiating posttraumatic neuropathological alterations, particularly those in which unexplained deficits persist after intracranial mass lesions have been removed. In our case report, for the first time, PET/CT offered microstructural and functional confirmation of KWNP. Moreover, our case suggests that 18 F-FDG PET/CT may serve as an important reference for the probability of functional recovery.

## Data Availability

Data are available on reasonable request from the corresponding author.

## Abbreviations

KWNP: Kernohan-Woltman notch phenomenon
MRI: Magnetic resonance imaging
18F-FDG: Fluorine-18 fluorodeoxyglucose
PET/CT: Positron emission tomography/computed tomography
TBI: Traumatic brain injury
